# SIRT3 inhibition suppresses hypoxia‐inducible factor 1α signaling and alleviates hypoxia‐induced apoptosis of type B spermatogonia GC‐2 cells

**DOI:** 10.1002/2211-5463.13523

**Published:** 2022-11-27

**Authors:** Zixuan Wang, Chunchun Zhu, Yangyang Song, Xiaoyun Chen, Jie Zheng, Lian He, Xing Liu, Zhu Chen

**Affiliations:** ^1^ University of Chinese Academy of Sciences Beijing China; ^2^ State Key Laboratory of Freshwater Ecology and Biotechnology, Institute of Hydrobiology Chinese Academy of Sciences Wuhan China; ^3^ Department of Reproduction, Maternal and Child Health Hospital of Hubei Province, Tongji Medical College Huazhong University of Science and Technology Wuhan China; ^4^ Health Science Center Yangtze University Hubei China

**Keywords:** apoptosis, GC‐2, HIF‐1α, hypoxia, SIRT3, spermatogonia

## Abstract

Hypoxia has been reported to be an important factor leading to male infertility, and it has been reported that hypoxia can induce the apoptosis of mouse spermatogenic cells. Sirtuin 3 (SIRT3) has been reported to promote the degradation of hypoxia‐inducible factor 1α (HIF‐1α), and thus, we hypothesized that SIRT3 may influence hypoxia‐induced apoptosis of spermatogonia. In this study, we overexpressed or inhibited SIRT3 in mouse type B spermatogonia GC‐2 cells and then subjected the cells to hypoxia or normoxia, before examining hypoxia‐responsive gene expression and cell viability. We report that SIRT3 stabilizes hypoxia‐inducible factor 1α (HIF‐1α) and activates its downstream target gene expression in GC‐2 cells. We also show that the SIRT3 inhibitor 3‐TYP suppresses HIF‐1α target gene expression and alleviates hypoxia‐induced apoptosis of GC‐2 cells. Our study reveals the critical role and underlying mechanisms of SIRT3 in hypoxia‐induced apoptosis of mouse type B spermatogonia GC‐2 cells.

## Graphical abstract

Hypoxia has been reported to be an important factor leading to male infertility. In this study, we report that NAD^+^‐dependent deacetylase, SIRT3, stabilizes hypoxia‐inducible factor 1α (HIF‐1α) and activates its downstream target gene expression in mouse type B spermatogonia GC‐2 cells. SIRT3 inhibitor 3‐TYP suppresses HIF‐1α target gene expression and alleviates hypoxia‐induced apoptosis of GC‐2 cells.

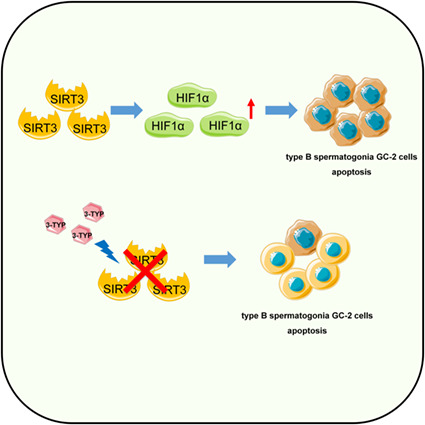

**Abbreviations**
HIF‐1α: hypoxia‐inducible factor 1α
NAD: nicotinamide adenine dinucleotide
PHD: prolyl hydroxylases
ROS: reactive oxygen species
SIRT: sirtuin

About 10–15% of couples in the world suffer from infertility, of which male infertility accounts for more than half [1]. Male infertility can be caused by many factors, including orchitis and varicocele [2, 3]. The pathogenesis of male infertility is usually caused by genetic and environmental factors [4]. Among environmental factors, hypoxia has been reported to be an important factor leading to male infertility [5]. It was reported that hypoxia can induce the apoptosis of mouse spermatogenic cells, and a hypoxia‐induced model of male infertility in rats was established [6, 7]. In addition, hypoxic conditions can also induce oxidative stress through the generation of uncontrolled reactive oxygen species (ROS) in mitochondria, posing a threat to cell survival [8, 9].

Hypoxia‐inducible factor 1α (HIF‐1α), as a transcription factor, plays critical roles in cell response to hypoxia, inducing target genes to function in various processes, including apoptosis and metabolic conversion from oxidative phosphorylation to anaerobic glycolysis [10]. The stability and transcriptional activity of HIF‐1α are mainly regulated by oxygen‐dependent hydroxylases. Two proline residues of HIF‐1α (proline 402 and 564) are hydroxylated by prolyl hydroxylases (PHD 1–3) under normoxic conditions. Subsequently, hydroxylated HIF‐1α is recognized by the VHL E3 ubiquitin ligase complex and modified by ubiquitination, which is then degraded by the proteasome. However, under hypoxia, hydroxylation is blocked and the accumulated HIF‐1α translocates to the nucleus, inducing downstream gene expression [11, 12, 13, 14].

SIRT3 is a member of the nicotinamide adenine dinucleotide (NAD^+^) dependent deacetylase family, comprising seven isoforms (SIRT1‐7) [15]. SIRT3 mainly localizes to the mitochondrial matrix and regulates cellular stress response and energy metabolism [16, 17]. The previous study shows that SIRT3 mediates metabolic reprogramming by promoting the degradation of HIF‐1α and functions as a tumour suppressor by suppressing the production of reactive oxygen species (ROS) and regulating HIF‐1α [18]. However, the function of SIRT3 in hypoxia‐induced spermatogenic cell apoptosis remains to be studied.

In this study, we found that SIRT3 functions as a positive regulator of HIF‐1α transcriptional activity. In a mouse type B spermatogonia GC‐2 cell line [19], overexpression SIRT3 increases the HIF‐1α protein level and induces its target gene expression. Furthermore, treatment of GC‐2 cells with the SIRT3 inhibitor 3‐TYP [20, 21, 22] downregulates HIF‐1α protein level and suppresses its target gene expression, protecting GC‐2 cells from hypoxia‐induced apoptosis. These results indicate that SIRT3 inhibition might enhance spermatogonia resistance to hypoxia stress.

## Materials and methods

### Reagents and antibodies

Anti‐Myc (#sc‐40) antibody was purchased from Santa Cruz Biotechnology (Dallas, TX, USA). Anti‐HIF‐1α (#36169) was purchased from Cell Signaling Technology (Danvers, MA, USA). Anti‐β‐actin (#AC026) was purchased from ABclonal Company (Wuhan, China). Dual‐luciferase reporter assay system (#E194A) was purchased from Promega (Madison, WI, USA). 3‐TYP (#S8628) and NAC (#S1623) were purchased from Selleck (Houston, TX, USA). FITC‐Annexin V Apoptosis Detection Kit I (#556547) was purchased from BD Pharmingen (San Diego, CA, USA). CM‐H_2_DCFDA (#C6827) and MitoSOX™ Red (# M36008) were purchased from Thermo Fisher (Waltham, MA, USA). Annexin V‐FITC Apoptosis Detection Kit (#C1062) was purchased from Beyotime (Shanghai, China).

### Cell culture

GC‐2 cells were obtained from the National Collection of Authenticated Cell Cultures, China, and cultured in Dulbeccos' modified eagle medium (DMEM) (HyClone, Logan, UT, USA) with 10% fetal bovine serum (FBS). The cells were grown at 37 °C in a humidified incubator containing 5% CO_2_. The cells were cultured under hypoxic conditions (1% O_2_, 5% CO_2_ and balanced with N_2_) by using the Ruskinn INVIVO2 I‐400 workstation (Ruskinn Technologies, Leeds, UK).

### Luciferase reporter assays

Cells were grown in 24‐well plates and transfected with various amounts of plasmids by VigoFect (Vigorous Biotech, Beijing, China), as well as with pCMV‐Renilla used as an internal control. After the cells were treated with or without hypoxia for 18–24 h, the luciferase activity was determined by the dual‐luciferase reporter assay system (Promega). Data were normalized to Renilla luciferase. Data are reported as mean ± SD, which are representative of at least three independent experiments, each performed in triplicate.

### Semi‐quantitative real‐time PCR (qPCR)

Total RNAs were extracted using RNAiso Plus (TaKaRa Bio., Beijing, China) following the protocol provided by the manufacturer. cDNAs were synthesized using the Revert Aid First Strand cDNA Synthesis Kit (Thermo Scientific). MonAmp™ SYBR^®^ Green qPCR Mix (high Rox) (Monad Bio., Shanghai, China) was used for semi‐quantitative RT‐PCR assays. The primers for semi‐quantitative real‐time PCR assays are listed below: Mouse‐Slc2a1‐RT‐F: 5′‐ATCCCAGCAGCAAGAAGGTGA‐3′; Mouse‐Slc2a1‐RT‐R: 5′‐TGGTGGATGGGATGGGCTCTCC‐3′; Mouse‐Vegf‐RT‐F: 5′‐ATGCCAAGTGGTCCCAGGCTGC‐3′; Mouse‐Vegf‐RT‐R: 5′‐ATCGGACGGCAGTAGCTTCGC‐3′; Mouse‐β‐actin‐RT‐F: 5′‐CCTGAGCGCAAGTACTCTGTGT‐3′; Mouse‐β‐actin ‐RT‐R: 5′‐GCTGATCCACATCTGCTGGAA‐3′.

### Western blot analysis

Total protein of the cells was extracted with RIPA buffer containing 50 mm Tris (pH 7.4), 1% Nonidet P‐40, 0.25% sodium deoxycholate, 1 mm EDTA (pH 8), 150 mm NaCl, 1 mm NaF, 1 mm PMSF, 1 mm Na_3_VO_4_ and a 1 : 100 dilution of protease inhibitor mixture (Sigma‐Aldrich, Steinheim, Germany). The cell lysates were separated by SDS/PAGE, transferred onto a polyvinylidene difluoride (PVDF) membrane (Millipore, Bedford, MA, USA), blocked with 5% (w/v) nonfat milk, probed with indicated primary antibodies (anti‐Myc antibody, Santa Cruz #sc‐40, dilution 1 : 2000; anti‐HIF‐1α antibody, Cell Signaling Technology #36169, dilution 1 : 1000; Anti‐β‐actin antibody, ABclonal #AC026, dilution 1 : 2000) and corresponding secondary antibodies [HRP Goat Anti‐Rabbit IgG (H + L), ABclonal #AS014, dilution 1 : 5000; HRP Goat Anti‐Mouse IgG (H + L), ABclonal #AS003, dilution 1 : 5000], visualized by ECL western blotting detection reagent (Millipore) and photographed by Fuji Film LAS4000 mini‐luminescent image analyzer. Image J software (National Institutes of Health) was used to quantify protein levels based on the band density obtained by western blot analysis.

### Lentivirus‐mediated gene transfer

HEK293 cells were transfected with pHAGE‐SIRT3 or the empty vector along with the packaging vectors pSPAX2 and pMD2G. The medium was changed with fresh full medium (10% FBS, 1% streptomycin–penicillin and 10 μm β‐mercaptoethanol) after 8 h. Viral supernatants were collected and filtered through 0.45‐μm strainer 40 h later. GC‐2 cells were infected by viral supernatant and selected by 1 μg·mL^−1^ puromycin for 2 weeks.

### Measurement of intracellular ROS level

GC‐2 cells were cultured under hypoxia as indicated. After treatment, the cells were trypsinized and then counted. Cells (1 × 10^6^) were incubated in PBS solution containing 1 μm of CM‐H2DCFDA (#C6821, Thermo Fisher) at 37 °C for 60 min. Remove the loading buffer. Cells were washed with PBS three times, followed by flow‐cytometric analysis.

### Measurement of mitochondrial ROS level

GC‐2 cells were cultured under hypoxia as indicated. After treatment, the cells were trypsinized and washed with PBS. Then, cells were incubated in PBS solution containing 5 μm of MitoSOX™ (# M36008, Thermo Fisher) for 10 min at 37 °C. Wash cells gently three times with PBS, followed by flow‐cytometric analysis.

### Detection of apoptotic cell

GC‐2 cells were cultured under normoxia or hypoxia at indicated hours. For flow cytometry analysis, the cells were harvested and washed twice with PBS and then resuspended in 1X Binding Buffer at a concentration of 1 × 10^6^ cells·mL^−1^. 100 μL of the solution (1 × 10^5^ cells) was transferred to a 5 mL culture tube; 5 μL of FITC‐Annexin V and 5 μL of PI were added. The cells were gently vortexed and incubated for 15 min at 25 °C in the dark. 400 μL of 1X Binding Buffer was added to each tube and then analyzed using Beckman CytoFLEX S within 1 h. The data were analyzed with cytexpert software (Beckman Coulter, Brea, CA, USA). Besides, the cells were stained with Annexin V‐FITC Apoptosis Detection Kit (#C1062, Beyotime) according to the manufacturer's instructions in a 6‐well plate and imaged under a florescent microscope Nikon TE2000‐U (Nikon, Tokyo, Japan).

### Statistical analysis

graphpad prism 7 software (GraphPad Software, San Diego, CA, USA) was used for all statistical analyses. Differences between experimental and control groups were determined by the unpaired two‐tailed Student's *t*‐test. *P* values < 0.05 were considered statistically significant. Statistical significance is represented as follows: *P < 0.05, **P < 0.01, ***P < 0.001, ****P < 0.0001.

## Results

### SIRT3 stabilizes HIF‐1α and promotes its transcriptional activity

**Fig. 1:**
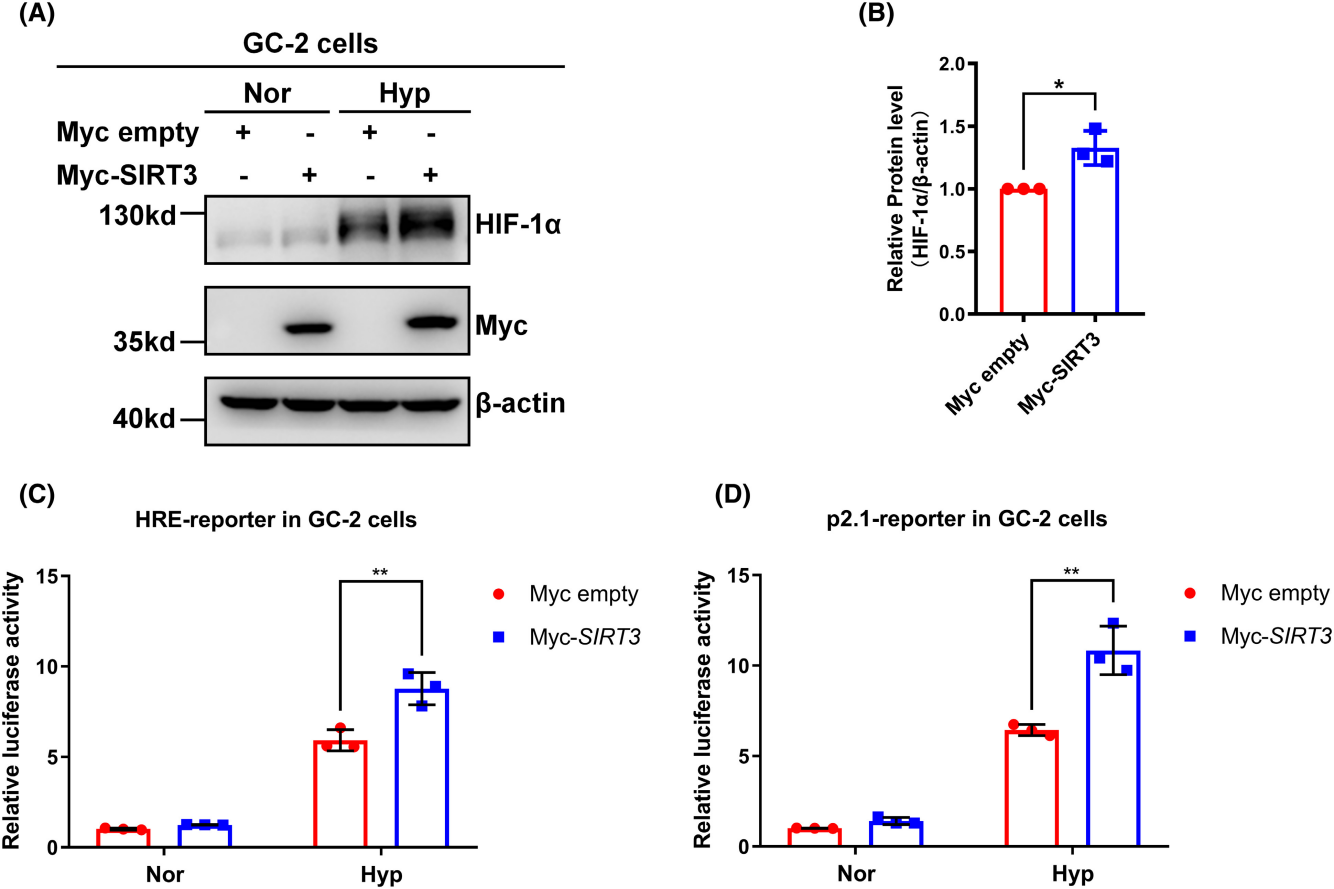
SIRT3 stabilizes HIF‐1α and augments its transcriptional activity in GC‐2 cells. (A) GC‐2 cells were transfected with Myc empty vector or Myc‐SIRT3 and then cultured under normoxia or hypoxia condition for 4 h. Total protein of the cells was extracted under indicated culture condition, and levels of endogenous HIF‐1α were examined by western blotting analysis. The experiment was repeated three times. (B) Quantitation of HIF‐1α proteins shown in (A). Data show mean ± SD; Student's two‐tailed *t*‐test. *P < 0.05. Data from three biologically‐independent replicates. (C) GC‐2 cells were transfected with HRE reporter, CMV‐Renilla as an internal control, together with Myc empty vector or Myc‐SIRT3 and then cultured under normoxia or hypoxia condition for 24 h. The cells were lysed under normoxia condition, and HRE reporter activity was analyzed by luciferase assay. Data show mean ± SD; Student's two‐tailed *t*‐test. **P < 0.01. Data from three biologically‐independent replicates. (D) GC‐2 cells were transfected with p2.1 reporter, CMV‐Renilla as an internal control, together with Myc empty vector or Myc‐SIRT3 and then cultured under normoxia or hypoxia condition for 24 h. The cells were lysed under normoxia condition, and p2.1 reporter activity was analyzed by luciferase assay. Data show mean ± SD; Student's two‐tailed *t*‐test. **P < 0.01. Data from three biologically‐independent replicates.

To evaluate the functional importance of SIRT3 in the hypoxia signalling pathway, we examined the effect of SIRT3 on the hypoxia‐induced protein level of HIF‐1α. In GC‐2 cells, hypoxia treatment dramatically increased the protein level of HIF‐1α, compared with normoxic conditions (Fig. 1A, lane 3 versus lane 1). Furthermore, overexpression of SIRT3 significantly enhanced the protein level of HIF‐1α (Fig. 1A,B). Then, we employed promoter assays to assess whether SIRT3 affects the transcriptional activity of HIF‐1α and used two well‐defined hypoxia‐inducible luciferase reporters, HRE reporter and p2.1 reporter. Upon hypoxia treatment, HRE reporter and p2.1 reporter activity was induced, which was enhanced by overexpression of SIRT3 (Fig. 1C,D).

### SIRT3 induces ROS accumulation and enhances hypoxia‐induced cell apoptosis.

**Fig. 2:**
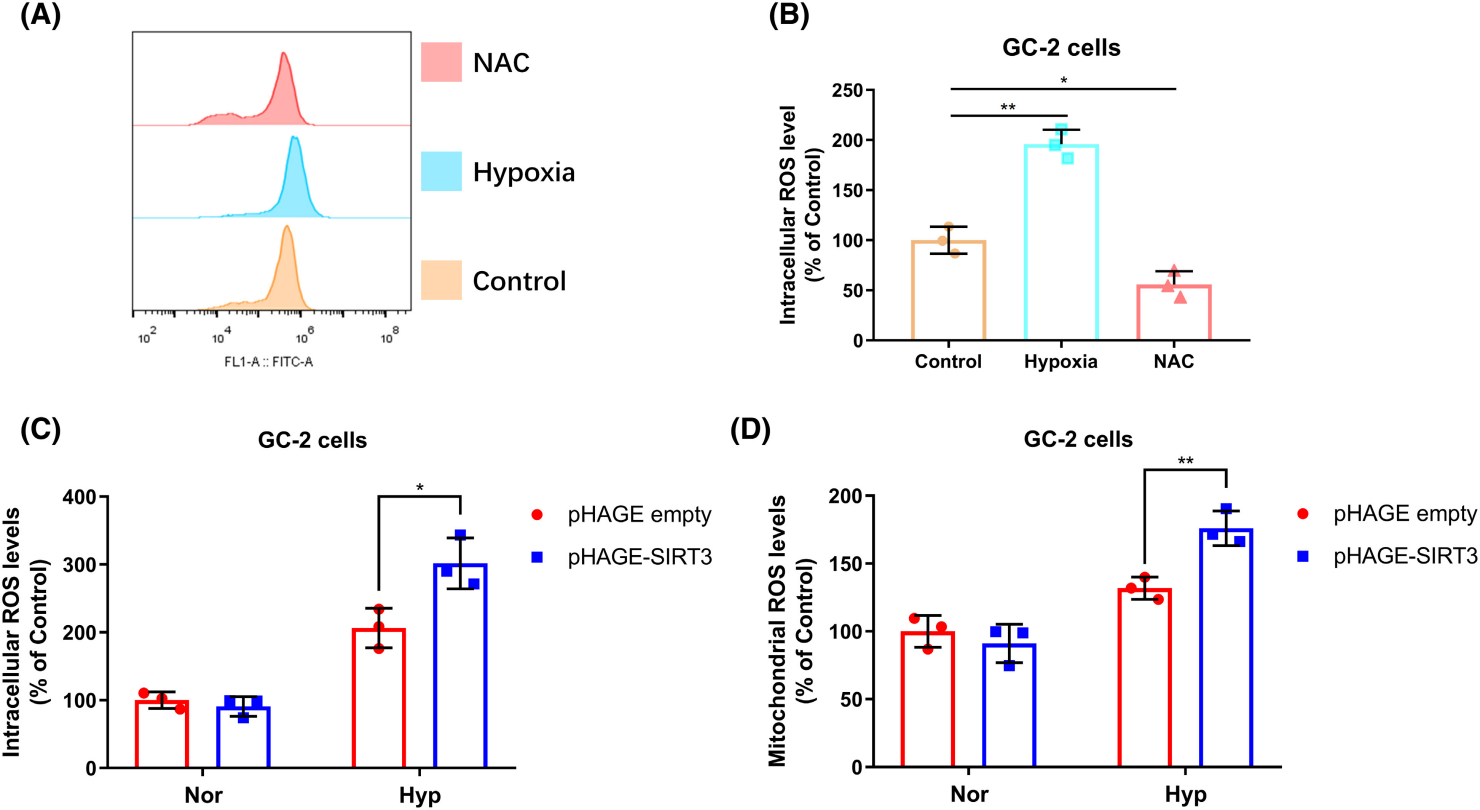
SIRT3 overexpression induces ROS accumulation under hypoxia. (A, B) GC‐2 cells were cultured under normoxia or hypoxia condition, or treated with NAC (20 mm) for 24 h. Then, the cells were harvested under indicated culture condition, and intracellular ROS levels were analyzed by flow cytometry. Data show mean ± SD; Student's two‐tailed *t*‐test. *P < 0.05, **P < 0.01. Data from three biologically‐independent replicates. (C) GC‐2 cells stably expressing SIRT3 or empty vector were cultured under normoxia or hypoxia for 24 h. Then, the cells were harvested under indicated culture condition, and intracellular ROS levels were analyzed by flow cytometry. Data show mean ± SD; Student's two‐tailed *t*‐test. *P < 0.05. Data from three biologically‐independent replicates. (D) GC‐2 cells stably expressing SIRT3 or empty vector were cultured under normoxia or hypoxia for 24 h. Then, the cells were harvested under indicated culture condition, and mitochondrial ROS levels were analyzed by flow cytometry. Data show mean ± SD; Student's two‐tailed *t*‐test. **P < 0.01. Data from three biologically‐independent replicates.

Many studies have reported that the cytotoxic ROS level is correlated with cell apoptosis upon hypoxia stress [23], and that aberrant control of mitochondrial ROS levels is a major factor contributing to apoptosis in cells exposed to prolonged hypoxia [24]. Consistent with these reports, intracellular ROS indeed could be induced by hypoxia stress (Fig. 2A,B). In addition, it could be inhibited by NAC (N‐acetyl‐L‐cysteine, a typical scavenger of ROS) [25] (Fig. 2A,B). Then, we examined the effect of SIRT3 on ROS accumulation. Hypoxia treatment significantly induced ROS accumulation, while SIRT3 overexpression upregulated the levels of intracellular and mitochondrial ROS by flow cytometry assay (Fig. 2C,D).

**Fig. 3:**
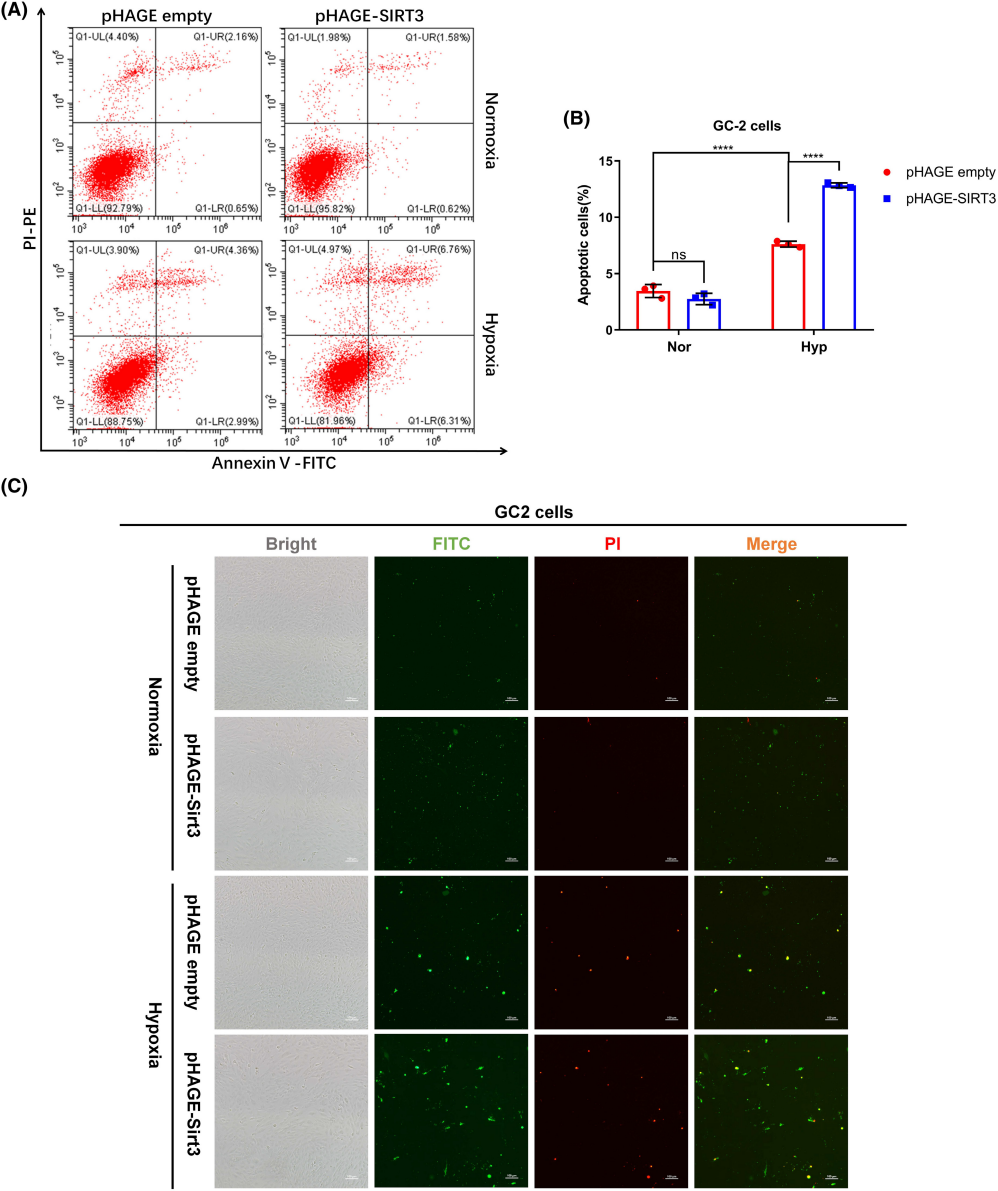
SIRT3 promotes hypoxia‐induced apoptosis in GC‐2 cells. (A, B) GC‐2 cells stably expressing SIRT3 or empty vector were cultured under normoxia or hypoxia for 48 h. Then, the cells were harvested under indicated culture condition, and apoptotic cells were analyzed by flow cytometry. Two‐way ANOVA analysis; Data show mean ± SD; Tukey's multiple comparisons test; ns—not significant, ****Adjusted *P* < 0.0001. Data from three biologically‐independent replicates. (C) GC‐2 cells stably expressing SIRT3 or empty vector were cultured under normoxia or hypoxia for 48 h. Then, the cells were fixed in 4% paraformaldehyde under indicated culture condition, and apoptotic cells were detected by fluorescence microscopy. Scale bar = 100 μm.

To determine the biological consequences of the transcriptional activity enhancement of HIF1α by SIRT3, we analyzed the effect of SIRT3 overexpression on cell apoptosis under hypoxia by Annexin V‐PI staining. Cells that are FITC‐Annexin V and PI negative are considered to be viable; cells that are FITC‐Annexin V positive and PI negative are considered to be in early apoptosis; and cells that are both FITC‐Annexin V and PI positive are considered to be in late apoptosis or already dead. Under normoxia, SIRT3 overexpression did not significantly affect apoptosis. However, hypoxia stress indeed induced cell apoptosis, and more apoptotic cells were detected in GC‐2 cells with SIRT3 overexpression, which was confirmed by flow cytometry assay (Fig. 3A,B). Furthermore, apoptotic cells were detected by fluorescence microscopy (Fig. 3C).

### SIRT3 inhibitor 3‐TYP regulates HIF‐1α protein stability and its downstream target gene expression.

**Fig. 4:**
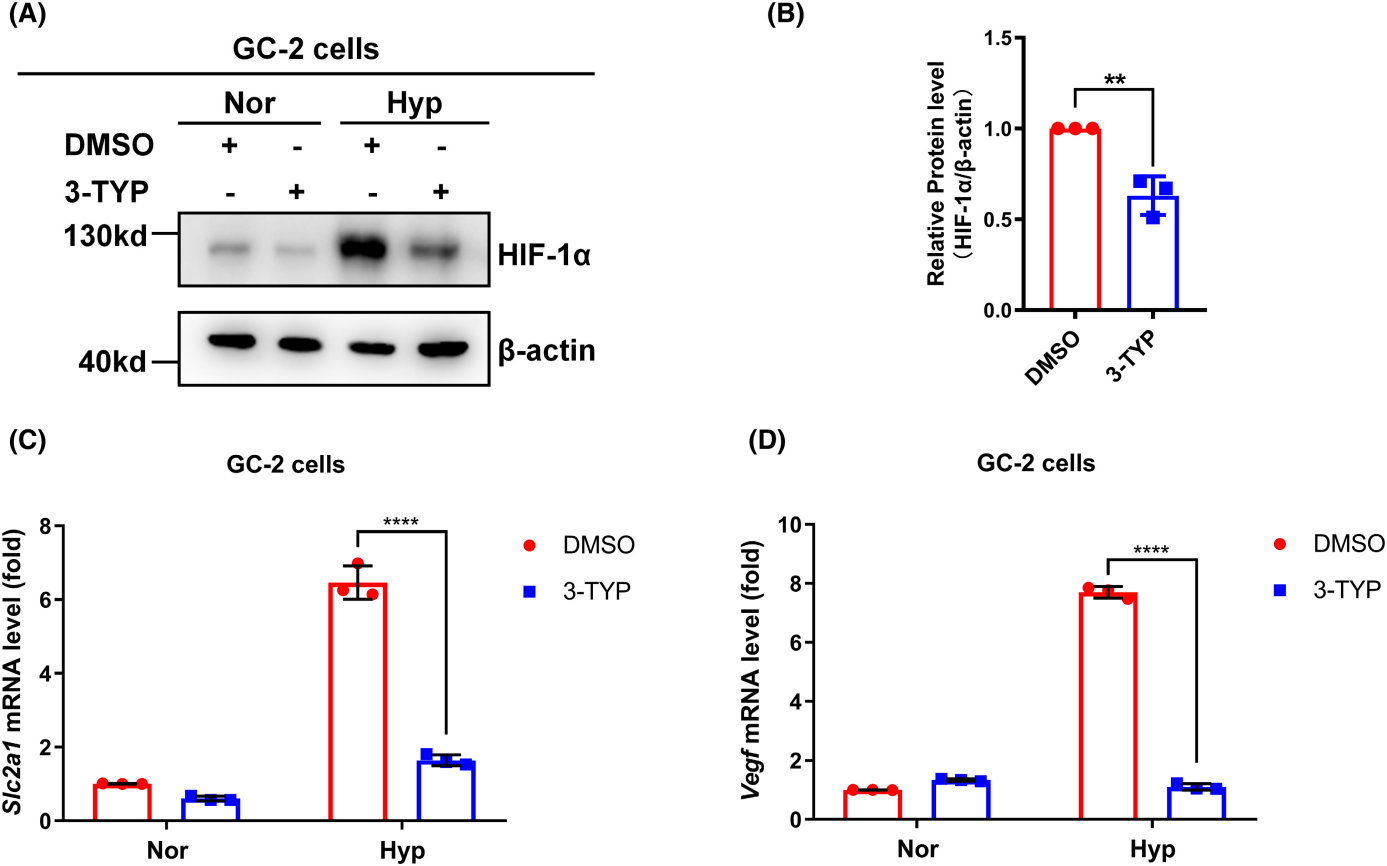
SIRT3 inhibitor 3‐TYP downregulates the protein level of HIF‐1α and suppressed the expression of its target genes in GC‐2 cells. (A) GC‐2 cells were treated with or without 3‐TYP (100 μm) for 24 h and then cultured under normoxia or hypoxia condition for 4 h. Total protein of the cells was extracted under indicated culture condition, and levels of indicated proteins were examined by western blotting analysis. The experiment was repeated three times. (B) Quantitation of HIF‐1α proteins shown in (A). Data show mean ± SD; Student's two‐tailed *t*‐test. **P < 0.01. Data from three biologically‐independent replicates. (C) GC‐2 cells were treated with or without 3‐TYP (100 μm) and then cultured under normoxia or hypoxia condition for 24 h. The cells were harvested under normoxia condition, and *Slc2a1* mRNA level was analyzed by qPCR analysis. Data show mean ± SD; Student's two‐tailed *t*‐test. ****P < 0.0001. Data from three biologically‐independent replicates. (D) GC‐2 cells were treated with or without 3‐TYP (100 μm) and then cultured under normoxia or hypoxia condition for 24 h. The cells were harvested under normoxia condition, and *Vegf* mRNA level was analyzed by qPCR analysis. Data show mean ± SD; Student's two‐tailed *t*‐test. ****P < 0.0001. Data from three biologically‐independent replicates.

To verify the role of SIRT3 in the regulation of the hypoxic response, we examined the effects of the SIRT3 inhibitor 3‐TYP in GC‐2 cells. Treatment with 3‐TYP suppresses the hypoxia‐induced protein level of HIF‐1α (Fig. 4A,B). We then examined the effect of 3‐TYP on HIF‐1α target gene expression. The mRNA levels of Slc2a1 and Vegf, two classical downstream target genes of HIF‐1α, were greatly induced under hypoxia, while 3‐TYP treatment significantly inhibited this induction (Fig. 4C,D).

### SIRT3 inhibitor 3‐TYP protects GC‐2 cells from hypoxia‐induced cell death

**Fig. 5:**
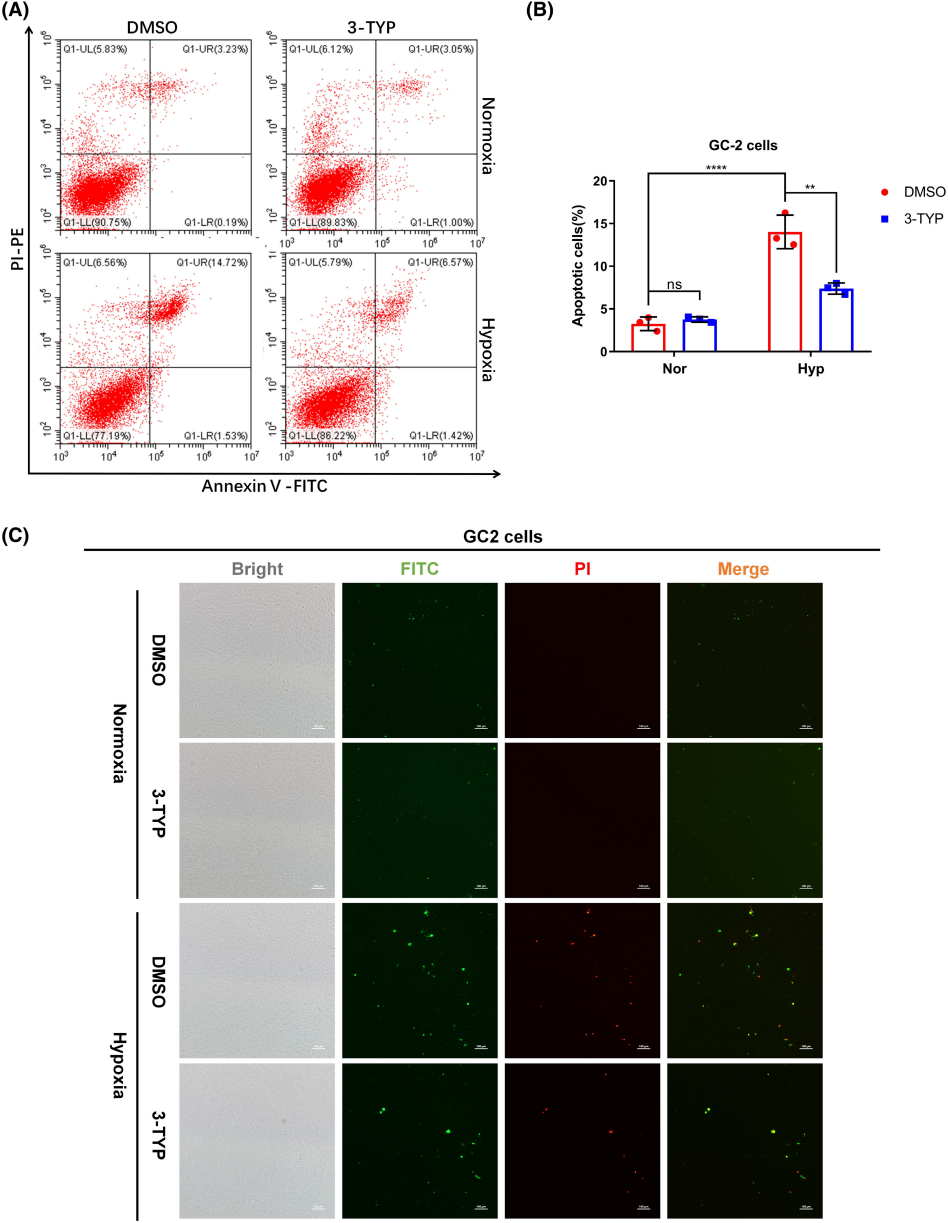
SIRT3 inhibitor 3‐TYP protects from hypoxia‐induced apoptosis in GC‐2 cells. (A, B) GC‐2 cells were treated with or without 3‐TYP (100 μm) and then cultured under normoxia or hypoxia for 48 h. DMSO was used as a control. Then, the cells were harvested under indicated culture condition, and apoptotic cells were analyzed by flow cytometry. Two‐way ANOVA analysis; Data show mean ± SD; Tukey's multiple comparisons test; ns—not significant, **Adjusted *P* < 0.01, ****Adjusted *P* < 0.0001. Data from three biologically‐independent replicates. (C) GC‐2 cells were treated with or without 3‐TYP (100 μm) and then cultured under normoxia or hypoxia for 48 h. DMSO was used as a control. Then, the cells were fixed in 4% paraformaldehyde under indicated culture condition, and apoptotic cells were detected by fluorescence microscopy. Scale bar = 100 μm.

To further elucidate the biological function of SIRT3 in hypoxia response in GC‐2 cells, we examined the role of the SIRT3 inhibitor 3‐TYP in hypoxia‐induced apoptosis by flow cytometry. Hypoxic treatment for 48 h could induce apoptosis of GC‐2 cells. However, compared with the control group, 3‐TYP treatment significantly protected GC‐2 cells from hypoxia‐induced apoptosis, which was confirmed by flow cytometry assay and fluorescence microscopy (Fig. 5A–C).

## Discussion

Previous studies have shown that an oxygen‐deprived environment reduces male fertility [6, 7]. Clinical studies and animal models have shown that hypoxia stress leads to decreased sperm count, sperm motility and testosterone levels. Apoptosis of spermatocytes is the main cause of spermatogenesis failure caused by hypoxia [7]. HIF‐1 has been reported to be one of the key mediators of asthenospermia by affecting sperm motility. In addition, the results in the GC‐2 cell model showed that hypoxia‐induced apoptosis of GC‐2 cells was mediated by HIF‐1α. Inhibition of HIF‐1α function can protect GC‐2 cells from hypoxia‐induced apoptosis [6, 26, 27].

Increasing evidence indicates that SIRT3 plays a vital role in the cellular response to various forms of stress, including DNA damage and hypoxia stress [28, 29, 30]. It has been reported that SIRT3 protects from hypoxia‐mediated cell death by maintaining mitochondrial membrane potential [30]. SIRT3 could reprogram cancer cell metabolism through HIF1α destabilization or suppress HIF‐1α by reducing mitochondrial ROS [18]. However, the regulation of HIF1α by SIRT3 in spermatogenic cells and the role of SIRT3 in hypoxia‐induced spermatogenic failure remain to be elucidated.

In this study, we found that SIRT3 stabilizes HIF‐1α and enhances its transcriptional activity in mouse type B spermatogonia GC‐2 cells. In addition, treatment of GC‐2 cells with SIRT3 inhibitor 3‐TYP disrupted the stability of HIF‐1α and inhibited its target gene expression. 3‐TYP treatment can protect GC‐2 cells from hypoxia‐induced apoptosis. These results contribute to understanding the role of SIRT3 in hypoxia response and the underlying mechanism of hypoxia‐induced apoptosis in GC‐2 cells and provide clues for the treatment of male infertility.

## Conflict of interest

The authors declare no conflict of interest.

## Author contributions

ZC and XL designed the experiments. ZW, CZ, YS, XC, JZ and LH performed the experiments and analyzed the data. ZC and XL wrote the manuscript. All authors discussed the results and commented on the manuscript.

## Data Availability

The data that support the findings of this study are available in Figs 1, 2, 3, 4, 5 of this article.
